# Optimization for the Production of Surfactin with a New Synergistic Antifungal Activity

**DOI:** 10.1371/journal.pone.0034430

**Published:** 2012-05-18

**Authors:** Xiangyang Liu, Biao Ren, Hong Gao, Mei Liu, Huanqin Dai, Fuhang Song, Zhenyan Yu, Shujin Wang, Jiangchun Hu, Chandrakant R. Kokare, Lixin Zhang

**Affiliations:** 1 Chinese Academy of Sciences Key Laboratory of Pathogenic Microbiology and Immunology, Institute of Microbiology, Chinese Academy of Sciences (CAS), Beijing, People’s Republic of China; 2 Institute of Applied Ecology, Chinese Academy of Sciences (CAS), Shenyang, People’s Republic of China; 3 Shengli Oilfield Xinhai Xingda Industrial Group Co., Ltd, Shandong, People’s Republic of China; 4 Graduate School, Chinese Academy of Sciences (CAS), Beijing, People’s Republic of China; 5 Bharati Vidyapeeth University, Erandwane, Pune, India; Belgian Nuclear Research Centre SCK/CEN, Belgium

## Abstract

**Background:**

Two of our long term efforts are to discover compounds with synergistic antifungal activity from metabolites of marine derived microbes and to optimize the production of the interesting compounds produced by microorganisms. In this respect, new applications or mechanisms of already known compounds with a high production yield could be continually identified. Surfactin is a well-known lipopeptide biosurfactant with a broad spectrum of antimicrobial and antiviral activity; however, there is less knowledge on surfactin’s antifungal activity. In this study, we investigated the synergistic antifungal activity of C_15_-surfactin and the optimization of its production by the response surface method.

**Methodology/Principal Findings:**

Using a synergistic antifungal screening model, we found that the combination of C_15_-surfactin and ketoconazole (KTC) showed synergistic antifungal effect on *Candida albicans* SC5314 when the concentrations of C_15_-surfactin and KTC were 6.25 µg/mL and 0.004 µg/mL, respectively. These concentrations were lower than their own efficient antifungal concentrations, which are >100 µg/mL and 0.016 µg/mL, respectively. The production of C_15_-surfactin from *Bacillus amyloliquefaciens* was optimized by the response surface methodology in shaker flask cultivation. The Plackett-Burman design found sucrose, ammonium nitrate and NaH_2_PO_4_.2H_2_O to have significant effects on C_15_-surfactin production. The optimum values of the tested variables were 21.17 g/L sucrose, 2.50 g/L ammonium nitrate and 11.56 g/L NaH_2_PO_4_·2H_2_O. A production of 134.2 mg/L, which were in agreement with the prediction, was observed in a verification experiment. In comparison to the production of original level (88.6 mg/L), a 1.52-fold increase had been obtained.

**Conclusion/Significance:**

This work first found that C_15_-surfactin was an efficient synergistic antifungal agent, and demonstrated that response surface methodology was an effective method to improve the production of C_15_-surfactin.

## Introduction

Biosurfactants (e.g., glycolipids, phospholipids, lipoproteins or lipopeptides, polymeric compounds, mycolic acids, and lipopolysaccharides) are a heterogeneous group of secondary metabolites with surface active properties, and described to be synthesized by a variety of bacteria [1], [2]. Surfactin is an important biosurfactant with superior surface activity and belongs to a group of cyclic lipoheptapeptides containing beta-hydroxyl fatty acids and D−/L- amino acid residues [3], [4]. Surfactins are mainly composed of three components: C_13_-surfactin, C_14_-surfactin, and C_15_-surfactin. Of those C_15_-surfactin has the highest: (1) surface activity, about 1000 times higher than the traditional chemical surfactant sodium dodecyl sulfate (SDS) [5], [6], and (2) hemolytic activity [7], [8]. C_15_-surfactin also has other activites, including anti-tumor, anti-microbial, and anti-mycoplasma functions [9], [10], [11], [12], [13]. Its amphiphilic structural characteristics contribute to its unique ability to interact with cell membranes and macromolecules such as enzymes and lipopolysaccharides (LPSs). Specifically, C_15_-surfactin non-competitively inhibits the activity of the alkaline phosphatase due to the chelating action by the free carboxyl groups of the Asp and Glu residues [14]. The binding of C_15_-surfactin with LPS inhibits the activity of LPS, which leads to the interruption of the LPS induced pathway [15], [16]. These properties demonstrate the commercial importance of C_15_-surfactin, specifically in the biomedical science and pharmaceutical fields [17], [18].

Yet, even with significant investigations on C_15_-surfactin, commercial production has been impeded by its high production cost due to low product yield. To address this problem, the discovery of an alternative C_15_-surfactin producer or improved methods for the efficient production of C_15_-surfactin is of particular importance. We have constructed a high quality microbial natural product library, from which *Bacillus velezensis* strain H3, *Saccharopolyspora* sp. A9 and *Streptomyces* sp. B3 have been recently identified to be biosurfactant producers [19], [20], [21]. This microbial natural product library has been a rich source for the discovery of C_15_-surfactin producing strains.

The goal of the current research is to demonstrate an efficient method for the production of C_15_-surfactin and the novel synergistic antifungal effects of surfactins with ketoconazole against *Candida albicans*. On the basis of this discovery, the medium composition was optimized to enhance the productivity of C_15_-surfactin by a novel marine derived *Bacillus amyloliquefaciens* strain MB199. This was achieved by combining the Plackett-Burman design (PBD), the steepest ascent design, and the central composite rotatable design (CCRD) of response surface methodology (RSM). This study will provide promising results for the development of new antifungal drug combination.

## Materials and Methods

### Synergistic Antifungal Assay

A synergistic antifungal assay was performed with surfactins according to the previously described method [22]. Briefly, *Candida albicans* SC5314 cells (∼1×10^4^) were inoculated in a final volume of 80 µL mixture of RPMI 1640 medium, 8% Alamar blue (BioSource International, Camarillo, CA), and 2 µL drugs in each well of flat bottom, 96-well microtiter plates (VWR, West Chester, PA). *Candida albicans* SC5314 cells was incubated overnight at 35°C, 80% humidity, and 5% CO_2_. Surfactins and KTC were prepared as stock solutions in DMSO. Growths of fungus cells were affected in the presence and absence of a sub-clinical concentration of 0.004 µg/mL ketoconazole. To determine the percentage of remaining viable cells, the fluorescence was measured at an excitation wavelength (Ex) of 544 nm and an emission wavelength (Em) of 590 nm using an EnVision 2103 multilabel reader (PerkinElmer, USA).

**Figure 1:**
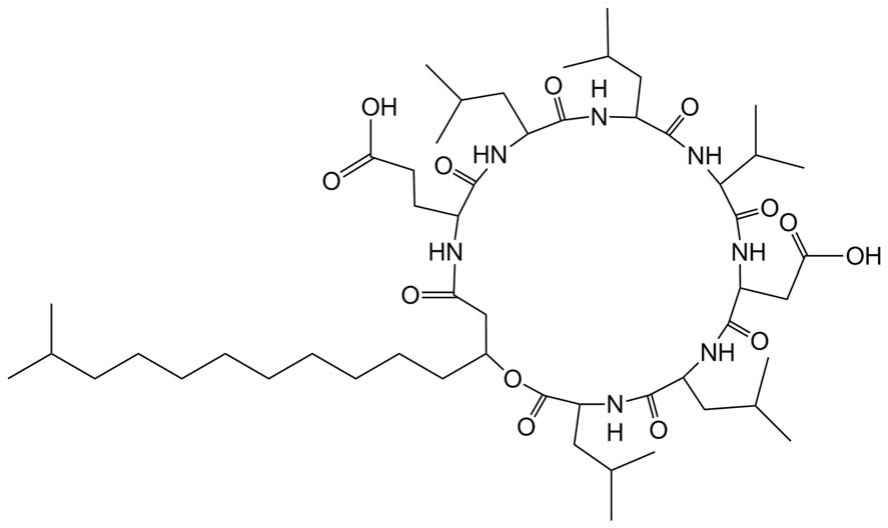
The structure of C_15_-Surfactin.

To determine the Minimum inhibitory concentrations (MICs), concentrations of surfactins were diluted by a serial 2-fold dilution method according to a modified protocol from the Clinical and Laboratory Standards Institute (formerly National Committee for Clinical Laboratory Standards) M-38A and M-27A2 methods. The MIC was defined as a concentration of an antimicrobial that prevented 100% of cell growth during 18-hr incubation at 35°C.

**Table 1:** The Plackett-Burman design for screening variables for C_15_-surfactin production.

Factors(g/L)	Code	Lowlevel(−1)	Highlevel(+1)	Coef*	*F*-value	*p*-value
**Sucrose**	***x*** **_1_**	**10**	**30**	−**9.98**	**26.10**	**0.0145**
**NH_4_NO_3_**	***x*** **_2_**	**0**	**4**	**8.07**	**17.06**	**0.0257**
K_2_HPO_4_·3H_2_O	*x*_4_	0.5	6	4.98	6.49	0.0841
**NaH_2_PO_4_·2H_2_O**	***x*** **_5_**	**5**	**15**	**8.55**	**19.16**	**0.0221**
MgSO_4_·7H_2_O	*x*_7_	0	0.4	−0.88	0.20	0.6828
MnCl_2_·4H_2_O	*x*_8_	0	0.004	2.48	1.62	0.2934
Yeast extract	*x*_9_	0.05	0.35	−4.61	5.57	0.0994
Temperature	*x*_10_	27	30	1.72	0.78	0.4433

R^2^ = 96.25%, R^2^ (adj)  = 86.25%.

* Coef: coefficient.

**Table 2:** The Placket-Burman design variables (in coded levels) with C_15_-surfactin yield as response.

Run	Variable levels											Yield of C_15_-surfactin (mg/L)
	*x*_1_	*x*_2_	*x*_3_	*x*_4_	*x*_5_	*x*_6_	*x*_7_	*x*_8_	*x*_9_	*x*_10_	*x*_11_	
1	1	−1	1	1	1	−1	−1	−1	1	−1	1	12.70±0.88
2	−1	1	−1	1	1	−1	1	1	1	−1	−1	53.00±0.29
3	1	−1	1	1	−1	1	1	1	−1	−1	−1	15.19±0.72
4	1	1	1	−1	−1	−1	1	−1	1	1	−1	3.92±0.42
5	−1	−1	−1	−1	−1	−1	−1	−1	−1	−1	−1	14.55±0.90
6	1	1	−1	−1	−1	1	−1	1	1	−1	1	12.73±0.99
7	−1	−1	1	−1	1	1	−1	1	1	1	−1	38.27±1.45
8	−1	1	1	−1	1	1	1	−1	−1	−1	1	51.72±1.69
9	1	−1	−1	−1	1	−1	1	1	−1	1	1	17.88±1.01
10	−1	−1	−1	1	−1	1	1	−1	1	1	1	23.22±0.99
11	1	1	−1	1	1	1	−1	−1	−1	1	−1	47.93±0.80
12	−1	1	1	1	−1	−1	−1	1	−1	1	1	49.31±0.18

**Table 3:** Design and results of CCD.

Run	Sucrose		NH_4_NO_3_		NaH_2_PO_4_·2H_2_O		Yield of C_15_-Surfactin (mg/L)
	Code *X_1_*	*X_1_* (g/L)	Code *X_2_*	*X_2_* (g/L)	Code *X_3_*	*X_3_* (g/L)	
1	−1	17	1	2.5	1	14	118.08±1.23
2	0	20	0	2.0	−1.68	17	45.56±0.42
3	1	23	1	2.5	1	14	130.41±12.94
4	0	20	0	2.0	1.68	17	100.60±1.26
5	0	20	1.68	2.8	0	10	128.96±2.38
6	1	23	1	2.5	−1	6	106.70±5.91
7	1	23	−1	1.5	−1	6	49.84±1.71
8	0	20	−1.68	1.2	0	10	96.07±5.94
9	−1.68	15	0	2.0	0	10	131.39±0.87
10	1.68	25	0	2.0	0	10	98.41±25.73
11	0	20	0	2.0	0	10	126.27±3.13
12	0	20	0	2.0	0	10	121.33±1.85
13	−1	17	1	2.5	−1	6	102.68±3.83
14	0	20	0	2.0	0	10	119.36±0.53
15	−1	17	−1	1.5	−1	6	85.18±10.36
16	1	23	−1	1.5	1	14	106.23±6.41
17	0	20	0	2.0	0	10	117.49±7.01
18	−1	17	−1	1.5	1	14	107.49±4.82
19	0	20	0	2.0	0	10	115.14±5.95
20	0	20	0	2.0	0	10	124.47±15.67

To determine whether drug interaction was synergistic, additive, or antagonistic for the combination of surfactins and KTC, fractional inhibitory concentration index (FICI) was used. FICI =  (MIC _drug A in combination_/MIC _drug A alone_) + (MIC _drug B in combination_/MIC _drug B alone_) [22]. The interaction was defined as synergistic if the FICI was <0.50, additive if the FICI was 0.50 to 4.0, and antagonistic if the FICI was >4.0.

**Table 4:** Synergy antifungal screening result^a^.

Samples	Anti-fungal MICs (µg/mL)	Synergistic anti-fungal MICs (µg/mL)
Acid Precipitation^b^	>100	50
Lipopeptide Mixture^c^	50	25
C_14_-surfactin	>100	12.5
C_15_-surfactin	>100	6.25
Cyclosporin A	>64	4

a The MIC of KTC is 0.016 µg/mL. The concentration of KTC in synergy antifungal screening experiment is 0.004 µg/mL, at which KTC does not show antifungal activity.

b Acid precipitation was obtained by centrifugation of cell broth at pH 2.0. It contains C13-surfactin, C14-surfactin, C15-surfactin and other kind of compounds.

c Lipopeptide mixture is purified fraction from acid precipitation, and is the mixture of C13-surfactin, C14-surfactin, and C15-surfactin.

### Micro-organisms and Culture Medium

Four microbial strains *Bacillus* sp. MB198, MB245, MB199, and MB200 were isolated from HuangBo Sea, China and shown using mass spectrometry to produce surfactins (data not shown). These strains were screened for the optimum surfactin producer with the fermentation medium as described in the following. The seed culture medium for all *Bacillus* strains was a Luria Bertani (LB) medium consisting of 5 g/L yeast extract, 10 g/L NaCl, 10 g/L tryptone, and a pH of 7.2. The fermentation medium used for shaker flask cultivation contained 20 g/L carbon source, 2.0 g/L nitrogen source, 3.0 g/L K_2_HPO_4_·3H_2_O, 10 g/L NaH_2_PO_4_·2H_2_O, 0.2 g/L MgSO_4_·7H_2_O, 0.002 g/L MnCl_2_·4H_2_O, and 0.2 g/L yeast extract. Carbon source and nitrogen source were arranged according to the description of the following section. Two milliliter of seed medium was transferred to a 250 mL Erlenmeyer flask containing 50 mL fermentation medium and incubated at 28°C for 48 h on a rotary shaker (200 rpm).

**Figure 2:**
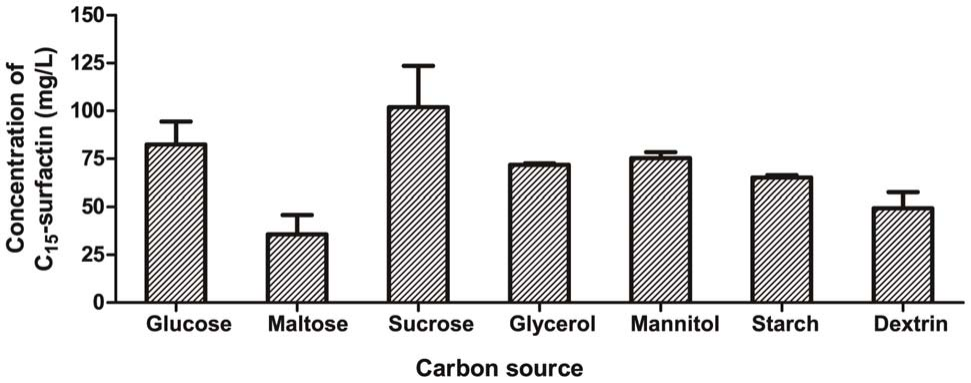
Effects of carbon source on the production of C_15_-surfactin from *B. amyloliquefaciens* MB199.

### Selection of Optimum Carbon Source and Nitrogen Source

To optimize the carbon source, glucose, sucrose, galactose, maltose, sucrose, glycerol, mannitol, soluble starch, and dextrin were evaluated. To optimize the nitrogen source, ammonium nitrate, ammonium sulfate, sodium nitrate, soybean flour, peptone, casein acid hydrolysate, urea, and glutamic sodium were evaluated. C_15_-surfactin (MW1035, Fig. 1) production was calculated by the method described in the section of “Analytical methods”.

**Figure 3:**
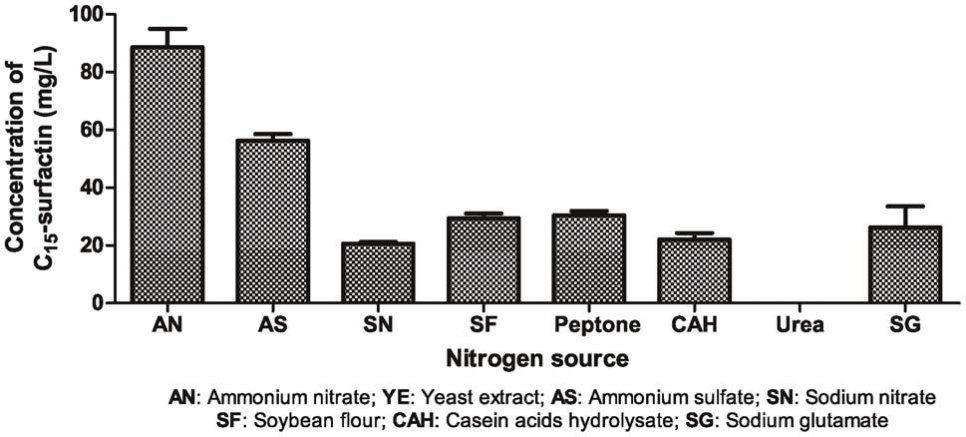
Effects of nitrogen source on the production of C_15_-surfactin from *B. amyloliquefaciens* MB199 with sucrose as the carbon source.

### 16S rDNA Sequence Analysis

Genomic DNA of *Bacillus* sp. was extracted using a TIANamp Bacteria DNA Kit (DP302, Tiangen Biotech (Beijing) Co., LTD., Beijing, China). The PCR method was performed according to the methods described previously [21]. *Bacillus* strains and calculations of sequence similarity were carried out using CLUSTAL X1 [23]. A phylogenetic tree was constructed using the neighbor-joining method and MEGA 4.0 software [24]. The topology of the phylogenetic tree was evaluated by 1000 bootstrap resampling replicates [25].

**Figure 4:**
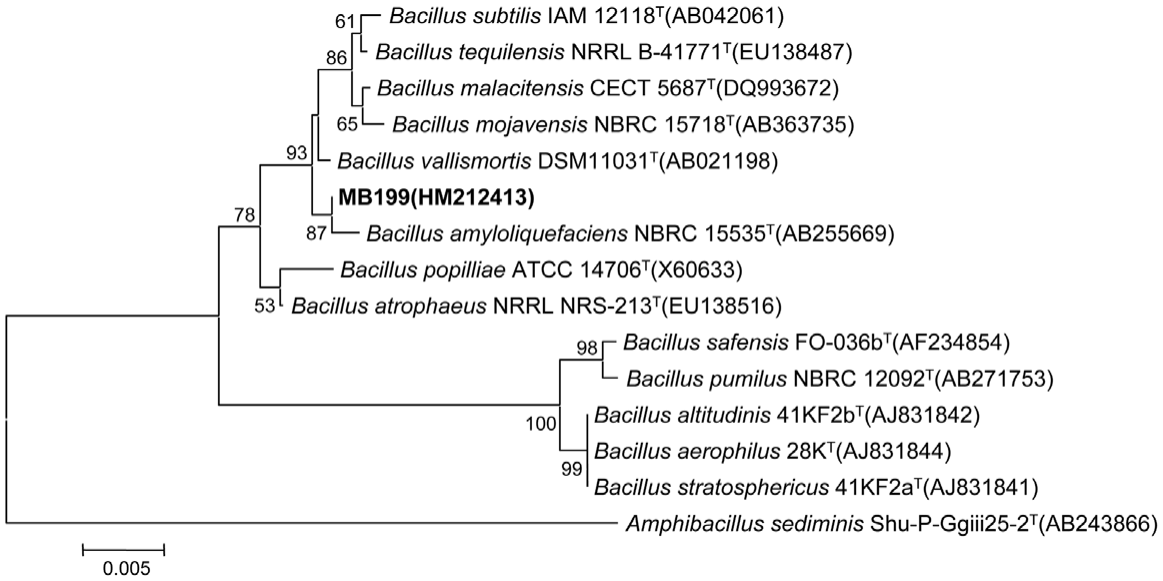
Neighbour-joining phylogenetic tree of *B. amyloliquefaciens* MB199 constructed by Mega 4.0. Numbers at nodes indicate levels of bootstrap support (%) based on a neighbour-joining analysis of 1000 resampled datasets; only values >50% are given. NCBI accession numbers are given in parentheses. Bar, 0.005 nucleotide substitutions per site.

### Experimental Design and Data Analysis

The most significant parameters affecting C_15_-surfactin production from *Bacillus amyloliquefaciens* MB199 were determined using the Plackett-Burman Design (PBD), as noted in Table 1 (Yeast extract is a good source of nitrogen and metals, so the low levels of x_2_, x_7_ and x_8_ could be set as 0). The analysis of C_15_-surfactin production was carried out in twelve experiments, each of which was performed two times (Table 2). Three dummy variables (x_3_, x_6_ and x_11_) were studied in 12 experiments to calculate the standard error. The final result was expressed as the average value of the two repeated experiments. The variables with confidence levels above 95% were considered to have significant effects on C_15_-surfactin production and were used for further optimization. Using the trends in the data provided by the results of the PBD, the experiments were adapted by increasing or decreasing the concentrations of each variable following the result of PBD [26]. Finally, a central composite rotatable design was produced with the values of each variable noted with the design matrix (Table 3). The low, middle, and high levels of each variable were designated as −1.68, −1, 0, and 1, 1.68, respectively and a response surface was produced.

**Table 5:** Design and results of path of steepest ascent experiment.

Run	Factor			Yield of C_15_-surfactin (mg/L)
	*X_1_* (g/L)^a^	*X_2_* (g/L)	*X_3_* (g/L)	
1	23	1.54	6.2	71.90±11.53
2	20	2	10	124.18±3.53
3	17	2.46	13.8	103.71±1.61
4	14	2.92	17.6	101.75±7.68

a *X_1_, X_2_ and X_3_* represent Sucrose, NH_4_NO_3_ and NaH_2_PO_4_•2H_2_O, respectively.

### Statistical Analysis

Design Expert (Version 7.0, Stat-Ease Inc., USA) was used to generate the experimental designs and perform subsequent regression analysis of the experimental data. The quality of the polynomial model equation was judged statistically using analysis of variance (ANOVA) to determine the coefficient of determination, R^2^. The statistical significance was determined using the *F*-test and significance of the regression coefficients was determined using the *t*-test.

**Table 6:** Regression coefficients and their significance for response surface model.

Term	Coef	Standard Error	*P*
Intercept	120.62	2.63	<0.0001
**X_1_** a	−**5.54**	**1.75**	**0.0099**
**X_2_**	**12.04**	**1.75**	**<0.0001**
**X_3_**	**15.40**	**1.75**	**<0.0001**
X_1_*X_1_	−1.64	1.70	0.3576
X_2_*X_2_	−2.48	1.70	0.1749
X_3_*X_3_	−**16.43**	**1.70**	**<0.0001**
X_1_*X_2_	**6.62**	**2.28**	**0.0159**
X_1_*X_3_	**5.30**	**2.28**	**0.0427**
X_2_*X_3_	−4.95	2.28	0.0554

a *X_1_, X_2_ and X_3_* represent Sucrose, NH_4_NO_3_ and NaH_2_PO_4_•2H_2_O, respectively.

### Extraction of Surfactins

Isolation and extraction of surfactins were performed according to the previously described method [27] with minor modifications. Briefly, after 48 hours of fermentation 35 mL cell broth was subjected to centrifugation at 8000 rpm for 5 min to remove the bacteria cells. The supernatant was then subjected to an acid precipitation with 6 M HCl by adjusting the pH to 2.0 and refrigerated at 4°C for 24 h. The precipitant was collected by centrifugation at 8000 rpm for 5 min followed by 24 h of lyophilization at −50°C. The lyophilized powder was extracted with 2 mL methanol for 4 h. Then the methanol extract was filtered using a 0.45 µm filter.

**Table 7:** ANOVA of regression model.

Source	DF^1^	Seq SS^2^	Adj MS^3^	*F*	*P*
Regression	9	10310.53	1145.61	27.48	<0.0001
Pure error	5	88.64	17.73		
Lack of fit	5	328.26	65.65	3.70	0.0886
Total	19	10727.43			

Determination of coefficient R^2^ = 0.9611; adjusted determination coefficient Adj R^2^ = 0.9262.

1 DF, Degree of freedom;

2 SS, sum of squares;

3 MS, mean square.

**Figure 5:**
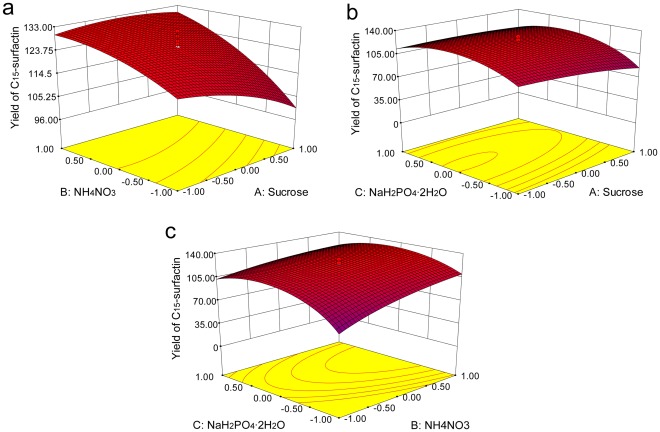
Response surface plot for C_15_-surfactin production by *B. amyloliquefaciens* MB199.

### Analytical Methods

The methanol extraction of surfactin was analyzed using a HPLC system (Agilent 1100 Series, CA, USA) equipped with an Agilent XDB C_18_ column (*Φ* 4.6 mm×15 cm, 5 µm). The mobile phase consisted of 90% methanol and 10% water (0.1% TFA). After loaded with 40 µL filtered methanol extract, and column was eluted at a flow rate of 1 mL/min. The elution was monitored by the UV absorbance at 210 nm. C_15_-surfactin was identified to be eluted at a retention time of 11.0 min according to LC-MS results.

**Figure 6:**
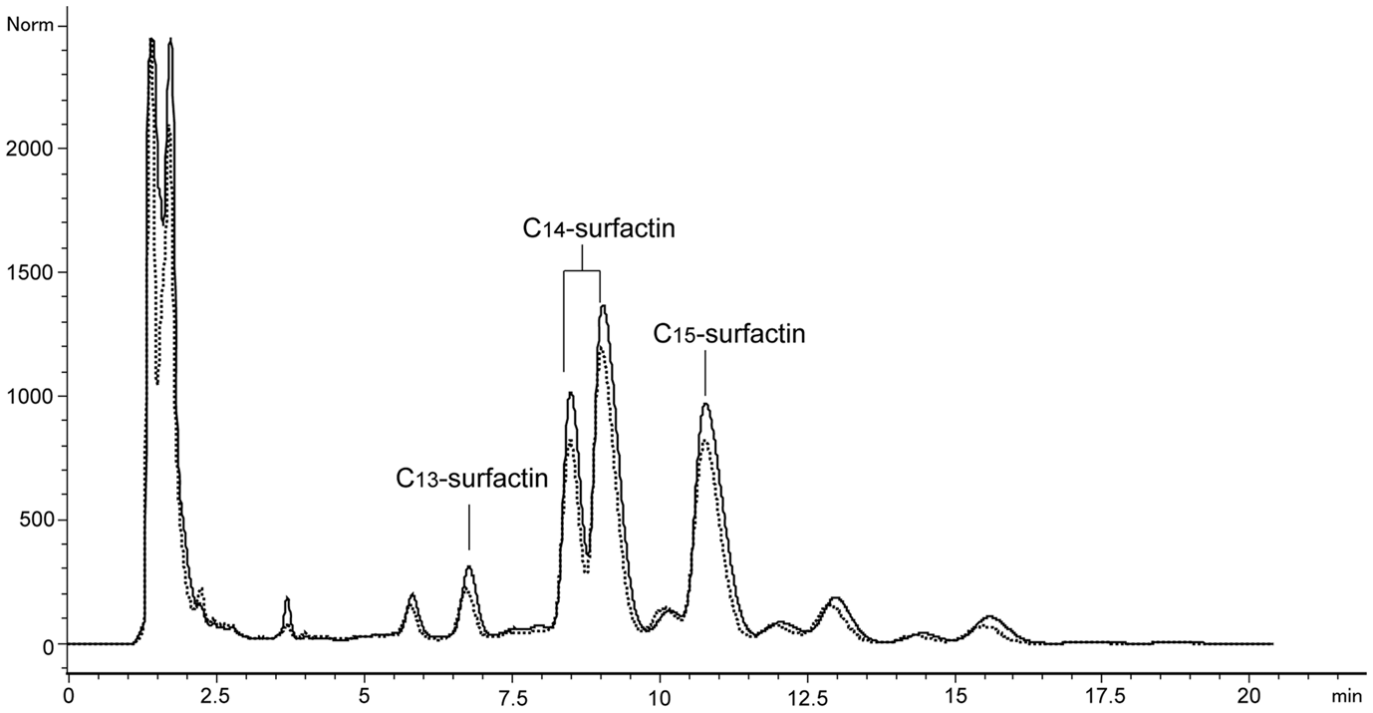
HPLC profile of surfactin produced by *B. amyloliquefaciens* MB199. The elution was monitored at 210 nm at a flow rate of 1 mL/min. The dashed line and the real line represent the HPLC profiles of surfactins produced in optimized and original culture media, respectively.

## Results and Discussion

### Synergistic Antifungal Activity of Surfactins

During the process of high throughput screening for the novel synergic antifungal compound, C_14_-surfactin and C_15_-surfactin were found to be most efficient when compared to the crude extract-acid precipitation (Table 4). It showed that C_14_-surfactin and C_15_-surfactin had synergistic antifungal activities with KTC against *Candida albicans* at 12.5 µg/mL and 6.25 µg/mL, respectively (Table 4). Based on these MIC values, the FICIs of C_14_-surfactin and C_15_-surfactin were less than 0.4 and 0.3 (all of them were less than 0.5), respectively, so the combination of surfactins and KTC were synergistic.

KTC is a frequently used antifungal drug (MIC = 0.016 µg/mL), which resulted in two drawbacks. One is the appearance of the resistant fungi. The other is the side-effect on human being produced by KTC at an efficient active concentration. So we design this synergy antifungal model to screen compounds which can synergize KTC with 1/4MIC (a concentration found to be no antifungal activity and also less side-effect on the patient). With this method C_15_-surfactin was identified to be best synergistic antifungal agent for KTC. This result not only proved the efficiency of our synergy screening model, but also highlights the new application of surfactin as a synergistic antifungal agent of KTC.

It has been reported that surfactin has synergistic activity with iturin on its hemolytic activity [28]. However, there is limited knowledge about the antifungal and synergistic antifungal activities of this compound. The results of our study showed a new effect of surfactin when KTC was incorporated. Surfactin has been known to interact with the cell membrane and disturbs the membrane’s stability [29], [30], [31], [32]. Among C_13_-surfactin, C_14_-surfactin and C_15_-surfactin, C_15_-surfactin was the most effective compound to interact with membranes because the longer fatty acid chain induces a greater interfacial activity of surfactins with the membrane [29]. This performance was helpful to explain our result that C_15_-surfactin was more active than C_14_-surfactin.

### Selection of Optimum Carbon Source, Nitrogen Source

The effect of the carbon source on C_15_-surfactin production of strain MB199 was given in Fig. 2. The optimum carbon source was found to be sucrose, which enabled MB199 to reach a maximum yield of 102.0 mg/L C_15_-surfactin.

With sucrose as the carbon source, the effect of the nitrogen source on C_15_-surfactin production for strain MB199 is given in Fig. 3. The maximum yield of surfactin (99.6 mg/L) was produced by the MB199 strain with ammonium nitrate used as the nitrogen source.

### Identification of Surfactin Producing *Bacillus* sp. MB199

The analysis of the 16S rRNA gene sequence (GeneBank accession no. HM212413) revealed that MB199 was similar to *B. amyloliquefaciens* NBRC 15535^T^ (similarity, 100%/1472 bps, based on 16S rRNA) (Fig. 4). Through the alignment and cladistic analysis of homologous nucleotide sequences of known *Bacillus*, phylogenetic relationships could be inferred. The approximate phylogenetic position of the strain is shown in Fig. 4. According to the gene sequence, the strain *Bacillus* sp. MB199 was identified as a strain of *B. amyloliquefaciens*, and named *B. amyloliquefaciens* MB199.

The selection of high amounts of surfactin producing strains is necessary for future mutation or bioengineering studies. Previously, our laboratory has constructed a high quality marine microbial natural product library containing novel microorganisms from marine environments. In this study, *B. amyloliquefaciens* was discovered to an efficient C_15_-surfactin producer from four *Bacillus* species. Actually, a recent report revealed that *B. amyloliquefaciens* is a producer of both lipopeptide and polyketide antibiotics [33]. These studies proved that *B. amyloliquefaciens* could be a promising strain used for the C_15_-surfactin production in the further engineering studies.

### Optimization of C_15_-surfactin Production by PBD

The importance of the eight parameters, namely, sucrose, NH_4_NO_3_, K_2_HPO_4_·3H_2_O, NaH_2_PO_4_·2H_2_O, MgSO_4_·7H_2_O, MnCl_2_·4H_2_O, yeast extract and temperature for C_15_-surfactin production was investigated by PBD. Table 1 shows the effects of these parameters on the response along with significant levels. Based on the statistical analysis, the parameters that significantly (confidence level >95%) affected the C_15_-surfactin production were sucrose, NH_4_NO_3,_ and NaH_2_PO_4_·2H_2_O with coefficients of (−) 9.98, (+) 8.07, and (+) 8.55, respectively. Other factors had no obvious effects and the low confidence levels indicating insignificant influence on the yield of C_15_-surfactin. With these significant parameters, the R^2^ was found to be 0.9625, which indicated the model could explain 96.25% of the total variations in the system.

### Optimization by the Path of Steepest Ascent Experiment

PBD results indicated that the effect of sucrose was negative, whereas those like NH_4_NO_3_ and NaH_2_PO_4_·2H_2_O were positive. Thus, decreasing sucrose concentration and increasing concentrations of NH_4_NO_3_ and NaH_2_PO_4_·2H_2_O should result in a higher production of C_15_-surfactin. For each of these factors, the average values from the PBD experiments were used as initial points for the path of steepest ascent experiments, and the concentrations were either increased or decreased as indicated by the PBD experiments. It showed the maximum production of C_15_-surfactin (124.18±3.53 mg/L) (Table 5). This was obtained when the parameters were 20 g/L sucrose, 2 g/L NH_4_NO_3_ and 10 g/L NaH_2_PO_4_·2H_2_O.

### Optimization by Response Surface Methodology

The data shown in Table 3 were analyzed using Design Expert software. The *t*-test and *P*-values were used to identify the effect of each factor on C_15_-surfactin production (Table 6), with a *P*-value of less than 0.05 indicating significance. Sucrose, NH_4_NO_3_ and NaH_2_PO_4_·2H_2_O had significant effects on C_15_-surfactin yield (*P*<0.05) and were able to explain 96.11% of the model variability. Therefore, the present prediction model reflected a good degree of correlation between the observed and predicted responses, implying the model was reliable for C_15_-surfactin production in the present study. The adjusted determination coefficient (R^2^ = 86.25%) was also satisfactory to confirm the significance of the model. The model can be shown as following:
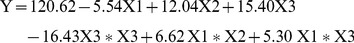
(1)Where Y is the predicted C_15_-surfactin yield, X1 is sucrose, X2 is NH_4_NO_3_, and X3 is NaH_2_PO_4_·2H_2_O.

Furthermore, the ANOVA analysis for the response surface quadratic model was presented in Table 7, which reported a statistically significant (*P*<0.05) regression at a 95% confidence level. To check the fitness of the polynomial model, the significant lack-of-fit was also reported in Table 7, which means there is some variation unaccounted for in the predicted model (the selected model does not well describe the data). In this study, *P* = 0.0886 indicated that the model was statistically insignificant lack of fit, so it was adequate for the prediction of C_15_-surfactin yield within the range of variables tested. The 3D response surface graphs provide a more complete representation of the effects of variables on the production of C_15_-surfactin (Fig. 5).

### Validation of the Optimized Condition

On the basis of medium optimization, the model predicted the maximum production of surfactin as132.61 mg/L, in the presence of 21.17 g/L sucrose, 2.50 g/L NH_4_NO_3_, and 11.56 g/L NaH_2_PO_4_·2H_2_O. To verify the predicted results, a validation experiment was performed in triplicate tests. Under the optimized condition, the observed experimental yield of average C_15_-surfactin was 134.2 mg/L, which is a 1.52-fold increase as compared to the yield in non-optimized media, suggesting that experimental and predicted values of C_15_-surfactin yield were in good agreement. This result therefore corroborated the predicted values and the effectiveness of the model, indicating that the optimized medium favors the production of C_15_-surfactin.

Actually, the pH value of the culture medium also has an effect on surfactin production. Surfactins have emulsification activities, so we use the emulsification index (EU/mL) as a parameter to evaluate the production of surfactins in cell broth. Our previous data showed that the production of surfactin will decrease in a culture medium with a pH less than 5 or larger than 9 [21]. In the present work, a pH of 7.2 was used throughout the optimization experiment. Noteworthy, the data on growth of *Bacillus amyloliquefaciens* was not measured in the present optimization experiment, so there was no information on the “specific productivity” of C_15_-surfactin by *Bacillus amyloliquefaciens*. However, a recent thesis showed that surfactin productivity was cell growth associated for *Bacillus subtilis* ATCC 21332 [34].

In the literature, a medium containing glucose (10.0 g/L) and ammonium nitrate (4.0 g/L) could lead to the highest quantity of surfactins (439.0 mg/L) by *B. subtilis* ATCC 21332 [35]. However, C_15_-surfactin was not clearly reported in these experiments due to the different analysis methods. Our present study focused on C_15_-surfactin production not only due to its effective biological activities, but also because purification of this compound was relatively easy by using HPLC (Fig. 6). In this respect, the present study was useful for the further investigations of the industrial production of C_15_-surfactin. Additionally, it has been found that surfactin producing *B. subtilis* strain S499 could produce a novel lipopeptide fengycin after the optimization of medium composition for the surfactin production [36]. In order to find out how the culture medium components influence the production of the other homologues of C_15_-surfactin, the HPLC profile of surfactins of *B. amyloliquefaciens* MB199 was measured. The results showed an increase in the yield of other homologues of C_15_-surfactin without influencing the diversity of the surfactins produced in the cell broth (Fig. 6).

In conclusion, the present work shows that C_15_-surfactin as a biomaterial could be utilized as a synergistic antifungal agent with ketoconazole for novel applications in biomedical and pharmaceutical fields. This study also offered a novel marine derived *B. amyloliquefaciens* strain MB199 which could efficiently produce C_15_-surfactin in shaker flasks. It showed that sucrose as a soluble carbon source and ammonium nitrate as a nitrogen source gave higher C_15_-surfactin production. The production of C_15_-surfactin was found to depend greatly on the key media components that were sucrose, ammonium nitrate, and NaH_2_PO_4_·2H_2_O. Using the RSM, it was possible to model individual and interactive effects of media and efficiently enhance the production of C_15_-surfactin.
